# Dataset and standard operating procedure for newborn screening of six lysosomal storage diseases: By tandem mass spectrometry

**DOI:** 10.1016/j.dib.2016.06.052

**Published:** 2016-07-05

**Authors:** Susan Elliott, Norman Buroker, Jason J. Cournoyer, Anna M. Potier, Joseph D. Trometer, Carole Elbin, Mack J. Schermer, Jaana Kantola, Aaron Boyce, Frantisek Turecek, Michael H. Gelb, C. Ronald Scott

**Affiliations:** a Department of Pediatrics, University of Washington, Seattle, WA 98195, USA; b PerkinElmer, Waltham, MA 02451, USA; c PerkinElmer, Turku 20750, Finland; d Department of Chemistry, University of Washington, Seattle, WA 98195, USA

## Abstract

In this data article we provide a detailed standard operating procedure for performing a tandem mass spectrometry, multiplex assay of 6 lysosomal enzymes for newborn screening of the lysosomal storage diseases Mucopolysaccharidosis-I, Pompe, Fabry, Niemann-Pick-A/B, Gaucher, and Krabbe, (Elliott, et al., 2016) [1]. We also provide the mass spectrometry peak areas for the product and internal standard ions typically observed with a dried blood spot punch from a random newborn, and we provide the daily variation of the daily mean activities for all 6 enzymes.

**Specifications Table**

**Table:** 

Subject area	Medicine, Biochemical Genetics, Newborn Screening
More specific subject area	Lysosomal storage diseases
Type of data	Figure, Table
How data was acquired	Tandem mass spectrometry with electrospray ionization
Data format	Analyzed
Experimental factors	Tandem mass spectrometry ion chromatogram peaks were integrated by computer
Experimental features	Tandem mass spectrometry ion chromatogram peak areas of quality control samples and random newborns
Data source location	Washington State Newborn Screening Laboratory, 1610 NE 150th St., P.O. Box 55729, Shoreline, WA 98155
Data accessibility	Data is available in this article

**Value of the data**

• Standard operation procedure gives full “hands-on” instructions for laboratory workers with appropriate training to carry out the 6-plex tandem mass spectrometry assay for lysosomal storage diseases.
• Raw data for the assays are provided so that other laboratories can compare their raw data to that given in this publication.
• Data is useful for setting up the new mass spectrometry assays in newborn screening laboratories including troubleshooting.

## 1 Data

**Fig. 1:**
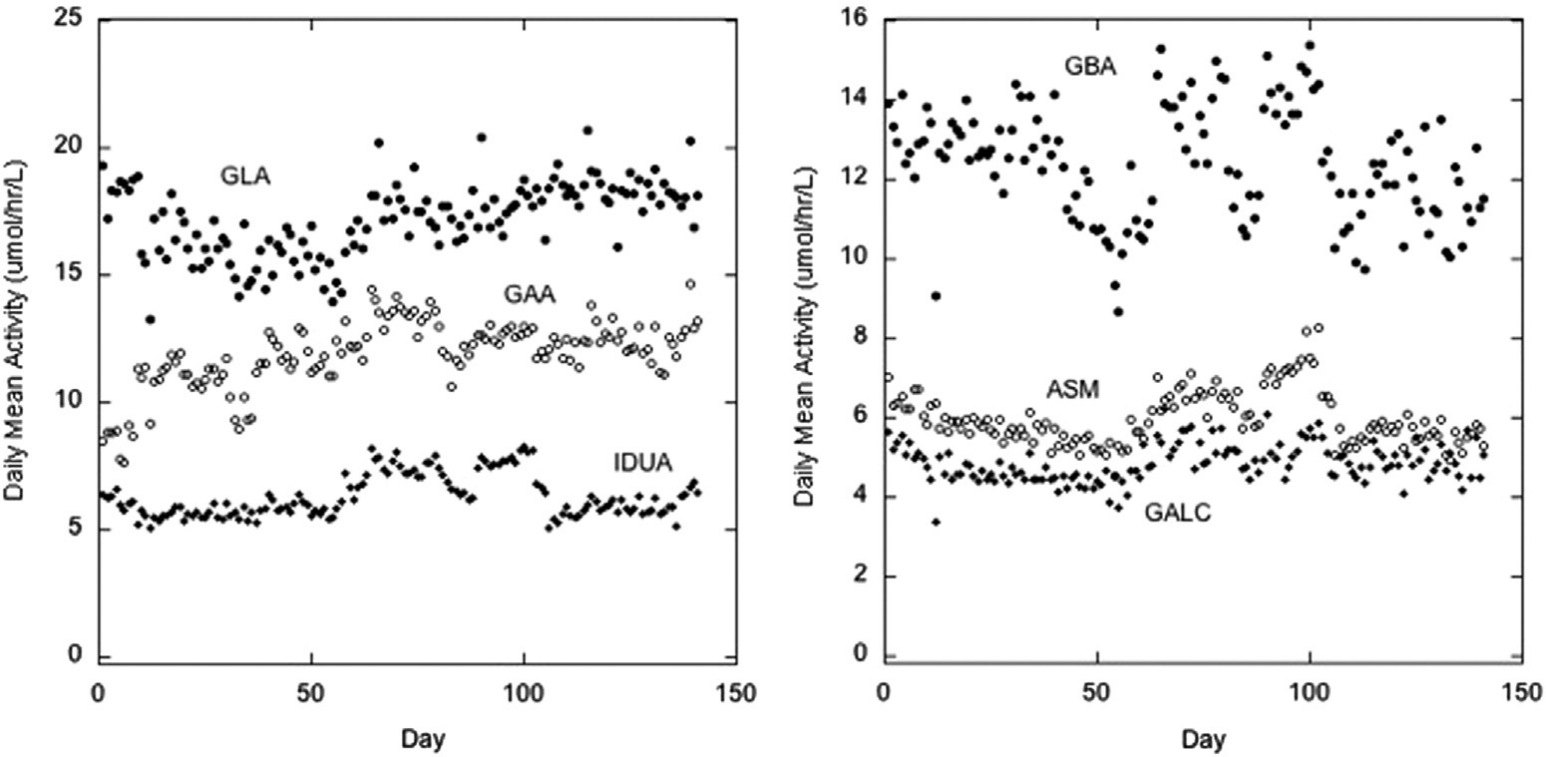
Daily variation of the daily mean enzymatic activity. The daily mean enzymatic activity for all newborn samples run on a single day is plotted versus the assay day.

Data provided are:

1) Fig. 1 provides the enzymatic activity (μmole/h/L blood) for each of 6 lysosomal enzyme activities averaged across all random newborn samples (data obtained according to the standard operating procedure given below). The mean activity is provided as a function of assay date.
2) Table 5 gives in peak areas for the multiple-reaction monitoring ion chromatograms for each of the 6 enzymatic products and internal standards observed with the quality control HIGH standard (typical of a healthy newborn).

### 1.1 Experimental design, materials and methods

#### 1.1.1 Standard operating procedure for the 6-plex assay

This standard operating procedure is the experimental details used to generate the data in this Data in Brief article as well as the previous publication [1], http://dx.doi.org/10.1016/j.ymgme.2016.05.015.

Note that the procedure below makes use of an incubator/shaker that accommodates only shallow well, 96-well plates. After incubation the solution is transferred to a deep-well, 96-well plate for subsequent liquid-liquid extraction. If your plate incubator can accommodate deep-well, 96-well plates, you can do the incubation of the DBS punch with assay cocktail in the deep-well plate as well as the ethyl acetate extraction in the same plate.

### 1.2 DBS NBS cards

DBS were prepared on two types of NBS cards, one made by Eastern Business Forms (made with Whatman 903 paper) and the other by PerkinElmer (226 cards, from Ahlstrom 226 filter paper).

### 1.3 6-Plex reagent preparation

#### 1.3.1 Preparation of 6plex buffer

Acarbose (MW 645.6), N-acetylgalactosamine (MW 221.2), and sodium taurocholate (MW 537.7) were obtained from CarboSynth Corp, and D-saccharic acid 1,4-lactone monohydrate (MW 210.1), succinic acid (MW 118.1), zinc chloride (MW 136.3), and sodium hydroxide were obtained from Sigma.

1. Dissolve 5.1 mg acarbose, 11.18 g N-acetylgalactosamine, 8.73 mg D-saccharic acid 1,4-lactone monohydrate, 14.80 g sodium taurocholate, 10.50 g succinic acid, and 82 mg of zinc chloride in nearly 1 L of purified water (Milli-Q, Millipore Corp. or other LC-MS/MS grade water).
2. Use a pH meter freshly calibrated with pH 4.0 and pH 7.0 buffers, and bring the pH of the mixture to 4.71 using sodium hydroxide. Finally, add water to bring to 1 L.
3. Store the buffer in a plastic bottle (PET or PP material) at +2 to +8 °C for up to 12 months.

#### 1.3.2 Substrate/internal standard mix

This is available form PerkinElmer (1 vial for ten 96-well plates). It contains a mixture of 6 substrates, 6 internal standards, and sodium oleate. The dried mix should be kept at −15 to −30 °C and can be stored for up to 1 year. Sodium oleate is a component to promote higher activity of the enzymes that act on sphingolipids. It is important to remember that it is supplied as a component of the substrate/internal standard mix, and thus is not added at the time of buffer preparation.

### 1.4 6-plex assay cocktail

1. Add 33 ml of the 6-plex buffer to one vial of 6-plex substrate/internal standard vial.
2. Sonicate for 20 min in a bath-type sonicator. Swirl and invert the vial several times. Do not vortex as this will produce a foam. A stir bar and stir plate can be used instead. Repeat sonication if necessary until all substrate is dissolved into a clear solution. Table 4 below gives the composition of assay cocktail.
3. Wrap in foil (light sensitive), store vial at room temperature for up to 1 week (or up to 1 month at +4 °C).

### 1.5 6-plex quench solution

Prepare in chemical hood. Add 500 mL of ethyl acetate to 500 mL of methanol, swirl to mix, store in hood at room temperature for up to 6 months in a glass bottle.

The quality of ethyl acetate used in this step as well as the liquid-liquid extract step (day 2 sample work-up) should be considered. Trace amounts of oxidizers (e.g., peracetic acid) from ethyl acetate manufacturing have been found to lower the product and internal standard intensities for GLA, GAA and IDUA. The lowering of these signals can affect the accurate measurement of low-activity samples. HPLC and LC-MS/MS grade ethyl acetate from J.T. Baker (Avantor) have been found to be routinely of good quality for this application (undetectable amount of oxidizers). Ethyl acetate with trace oxidizer contamination can cleaned by treatment with anion exchange resin (such as Dowex-1). If needed you can swirl 10 g of Dowex-1 in an Erlenmeyer flask with 50–100 mL of ethyl acetate, then decant, repeat 3 times (this is to remove contaminants which may be present on the surface of commercial Dowex-1). Then transfer the washed Dowex-1 to a glass bottle of ethyl acetate (1–2 L), swirl briefly, and then use for the assay. There is no need to remove the Dowex-1 beads, they will remain at the bottom of the bottle.

Methanol used should be LC-MS/MS grade.

### 1.6 6-plex mobile phase 84% acetonitrile/16% water/0.1% formic acid

1. Measure out 320 mL of HPLC grade water (Fisher Optima Grade) in a graduated cylinder then transfer to a 2 L volumetric flask.
2. In a chemical hood, add 2 mL of HPLC grade formic acid (Fisher Optima Grade) to the volumetric flask, then swirl to mix.
3. In a chemical hood, QS the volumetric flask to 2 L with HPLC grade acetonitrile (Fisher Optima Grade).
4. Invert to mix.
5. Store at room temperature for up to 3 months.

### 1.7 6-Plex assay protocol

#### 1.7.1 Incubation – day one

The layout of the 96-well plate is as follows:

filter paper blank: wells A1, B1, H1, A12, B12, H12
QC-Low (CDC): wells C1, C12
QC-High (CDC): wells D1, D12
QC-Low (PerkinElmer): wells E1, E12
QC-HIGH (PerkinElmer): wells F1, F12
QC-adult blood: wells G1, G12
random newborns: all remaining wells

1. Inspect all wells of the shallow well plates (0.5 mL, Nunc, Thermo scientific, cat. No. 267245) containing 3.2 mm DBS specimen (blood ~3.2 μL), and make note of any wells that contain a white spot (filter paper) or blood spot that is insufficient for testing.
2. Add the following DBS controls including 3 filter paper blanks; CDC QC LOW, CDC QC High, PE QC low, PE QC high, and QC CRS to columns 1 and 12. CDC QC DBS are available from the Centers for Disease Control and Prevention, and PE QC DBS are available from PerkinElmer. In the future, we will proceed with QC DBS from just a single supplier so that more newborn samples can be run per plate.
3. Fill a small narrow trough with approximately 10 mL of 6-plex assay cocktail. This will be enough for 3 plates.
4. To each well, add 30 μL assay cocktail using a multi-channel pipette. Place the tip of the pipette against the inside wall of the well when dispensing to allow cocktail to slide down the wall of the well for accurate delivery.
5. Seal plate with aluminum sealing film (StarSeal sealing tape aluminum foil, Star Lab Cat. E2796-9792, Hamburg, Germany), press firmly to ensure each well is sealed or liquid will be lost due to evaporation during incubation. You can use a sealing roller. These sealing films are not sold in the USA, but we have tested Axygen foil covers (Cat. PCR-AS-200 or VWR Cat. 47734-817), which work fine.
6. Place plates in the PerkinElmer Trinest incubator and incubate for 18 h (±15 min) at 37 °C±0.5 with orbital shaking at 400 rpm. Make note of start time and temperature on the assay logsheet.

### 1.8 Workup – day two

#### 1.8.1 Quenching and mixing

In our lab we use a 96-channel, manually operated pipette device (Rainin Liquidator) capable of liquid delivery to 96 wells of a 96-well plate in one trigger operation. You can also use other types of multi-channel pipettes, i.e. 12-channel, etc.

1. Quench and Transfer step should be done together for each plate.
2. Remove the seal from the plate and place the plate on Rainin liquidator platform.
3. Remove the stopping post from the liquidator on the side you placed the sample plate.
4. Load liquidator with LQR-200 filter tips, and fill the trough with 6-plex quench solution which is methanol/ethyl acetate (1:1).
5. Place an appropriately labeled deep well plate (Nunc, Thermo Scientific, cat no 260252) on the liquidator.
6. Collect 100 μL of quench solvent from the trough, move liquidator head to sample plate and dispense the 100 μL from the tips into the samples wells.
7. Mix the sample with quench solvent as follows: press tips against side wall of sample well when entering the sample well. You will have to pull the plate towards you using the liquidator plate platform. When the tips reach the bottom of the well push plate away from you. This action uses the tips to move the DBS punch to the side wall of the sample well and out of the way of the pipette tip.

Aspirate 100 μL of the sample volume up and down into the liquidator tips 10×.

### 1.9 Transfer sample solution to deep well plate

1. The liquidator volume draw can be left at 100 μL. Press tips against side wall of sample well when entering the sample well. You will have to pull the plate towards you using the liquidator plate platform. When the tips reach the bottom of the well push plate from away from you. This action uses the tips to move the DBS filter paper punch to the side wall of the sample well and out of the way of the pipette tip.
2. Aspirate all the sample volume into the liquidator tips.
3. Lift the liquidator head and tips out of the wells and ensure no DBS filter paper punches are stuck to the tips.
4. Dispense all the sample volume to an appropriately labeled, corresponding deep well plate. Some sample volume (in ethyl acetate) clings to the side walls of the tips. Wait an additional 10–15 s to allow the residual ethyl acetate in the tips to fall to the bottom of the tip and expel the remaining volume into the wells a second time.
5. The above steps are repeated to acquire the last 30 μL of volume in the samples wells. Try not to pull to much air into tips.

### 1.10 Ethyl acetate extraction, perform in chemical hood

1. Set up the liquidator volume dial to 200 μL.
2. Add ethyl acetate to the Liquidator reservoir.
3. Dispense a volume of 400 μL of ethyl acetate to each well for each plate.
4. One box of tips can be used for all sample plates as long as there was no contamination of the tips.
5. Replace the liquidator reservoir with HPLC grade water.
6. Dispense a volume of 200 μL of water to each well of each plate.
7. Mixing; Using the liquidator, quickly aspirate 200 μL of sample volume up and dispense down 20 times in each well. This is a critical step to ensure entire sample volume was miscible for a short period of time, and thus the sample is extracted from the water layer into the ethyl acetate layer. The two layers will separate out quickly. Use a new box of tips for each plate.
8. Seal the plates with self-adhesive foil (same type of adhesive foil used for incubation).
9. Centrifuge the plates at 2500 rpm for 5 min at room temperature to separate solvent layers.
10. After centrifugation, 100 μL of the upper layer of ethyl acetate phase is transferred from the deep well plate into a fresh shallow well plate (Nunc, Thermo Scientific) using the liquidator. A stopping post is used to stop the liquidator head from lowering the tips below the interface between the top and bottom solvent layers. Place the 6-plex assay post (84–85 cm length) on the left platform where you pull the samples from.
11. Place the deep well plate containing samples in ethyl acetate on the left plate platform of the liquidator.
12. Place an appropriately labeled, corresponding shallow well plate in the front of right plate holder, and load the Liquidator head with tips.
13. Squeeze handle on the liquidator before placing the tips into sample so air is expelled and does not disturb the separation of the two liquid layers.
14. Push liquidator head with tips, down into samples until it rests on stopping post.
15. Pull up 100 μL of the top layer (ethyl acetate) into the tips.
16. Transfer and dispense liquid into the corresponding shallow well plate on the right. Move quickly to the right plate as ethyl acetate has low viscosity and can drip from the tips.
17. Evaporate the solvent from the shallow plates with jets of oil-free air using a SPE 96 Dual Dryer – flow rate 60–40 L/min, temperature 35 °C, drying time 10±5 min.

Note, if you don not use the Liquidator you can obtain from PerkinElmer (jason.cournoyer@perkinelmer.com) a plastic multi-well spacer with holes that is placed on top of the deep well plate. The pipet tips on the multi-channel pipet are inserted into the holes in the spacer and lowered until the tips stop in the spacer. In this way the tips are lowered into the ethyl acetate layer but not into the lower water layer. It is important to use the proper tips as recommended by PerkinElmer. In this way, the tips are placed deeply enough into the ethyl acetate layer such that no air or water are drawn in the tips while 100 µL of ethyl acetate is drawn into the tips. If you don׳t have this spacer, you can do a trial liquid transfer where you use the indentation marks on the pipet tips to gauge how far the tips are inserted into the deep well plate. Also, an automated liquid handler can be set to be used for this step.

### 1.11 Preparation for MS analysis

1. Reconstitute dried sample residue with 200 μL of 6-plex mobile phase solution (84% acetonitrile/16% water/0.1% formic acid)
2. Cover each plate with non-adhesive aluminum foil.
3. Shake each plate for 10 min in the PerkinElmer Trinest incubator without heat and at a speed of 400 rpm to dissolve the residue. Keep plates level to prevent cross-well contamination.
4. See the next section on preparing the mass spectrometer for sample analysis before injecting samples.
5. Inject 15 μl per sample using a 10 μl loop. The effective volume of sample delivered is 10 μL as the loop is overfilled. Needle wash and flow-injection conditions for the autosampler are given below.
6. Samples are injected on the MS toward the end of day two and throughout the night.

### 1.12 Mass spectrometry

We have tested the 6-plex assay successfully on 5 types of instruments: Acquity TQD, Xevo TQD and Quattro Micro from Waters and the 3200 and 4000 from Sciex. The assay requires low-energy source conditions in order to minimize in-source fragmentation of the excess substrate that remains in the sample that is injected into the MS system. Under high-energy conditions, the substrate can breakdown to generate the enzymatic product and therefore can increase the measured activity in blanks and samples. Despite blank subtraction from DBS samples, it has been found that this in-source decay of the excess substrate can affect the accurate measurement of low activity samples and therefore these low-energy sources conditions are required for optimum assay performance. Even with this contribution to the blank, the ratio of assay response for the quality control high DBS (typical of a healthy newborn) to response from the no-blood blank is an order-of-magnitude higher than the analogous ratio for fluorimetric assays with 4-methylumbelliferyl substrates as discussed in the main text.

The necessary low-energy conditions can primarily be achieved by lowering the temperature in the source, but also by lowering entrance voltages (i.e., cone voltages for Waters instruments and declustering potentials for Sciex instruments) and the capillary voltage if necessary. See Table 2 below for examples of low-energy source settings for the Waters and Sciex instruments that were used successfully for the assay. The final settings should be determined using blanks and obtaining the lowest signal for product (from in-source fragmentation in blanks) but also keeping the IS signal as high as possible.

As mentioned, in addition to low source temperature, the optimized entrance voltages for the MRMs can be lowered to decrease the apparent activity of the blanks. This approach has shown to be useful for keeping GBA and GALC blanks low. Also, in some cases, lowering the capillary voltage has been found to be useful for keeping GLA blanks low.

The entire analytical method uses the MS/MS settings in Table 1, MRM transitions in Table 2, and inlet and autosampler settings in Table 3.

Flow injection flow rate method

**Table:** 

Time (min)	Flow rate (mL/min)	Curve
Initial	0.10	6
0.05	0.05	6
0.70	0.05	6
0.80	0.10	6
1.00	0.10	6

Routine cleaning of the Waters Mass Spectrometer.

We use a MS/MS electrospray source cleaning procedure daily to minimize source loading.

This applies to the Waters TQD instrument, but analogous procedures are used for other MS/MS instruments.

1. Remove previous day׳s cone. Remove probe and set on bench.
2. Thoroughly wipe out spray chamber with methanol, training may be required by your Waters service representative.
3. Rinse outside of probe in methanol, and gently wipe off. Be sure not to twist or this will move the capillary position and affect results.
4. Replace probe and insert cleaned cone (see below for cleaning instructions).
5. Using the 2777C software, rinse the injection syringe with wash 1 (methanol), 9× times.
6. Set up a plate with well #1 full of methanol. Run 3–4 injections of this methanol.
7. Switch to the 6-plex pump method. This method uses the 6-plex mobile phase.
8. Run the pump at 1.0 mL/min for 2–5 min to flush the system with the 6-plex mobile phase. Pressure will spike to about 1000 psi as the solvent is flushed out. When the solvent line is equilibrated with acetonitrile the pressure will drop.
9. Using the 2777C software, rinse the injection syringe with wash 2 (6plex mobile phase) 9× times.
10. The instrument is ready for sample analysis.

Cleaning the cone.

First Sonication:

1. Place inner cone and outer cone in beaker and pour approx. 20 mL of formic acid directly over cones. Make sure not to damage cone tip.
2. Add 10–20 mL of purified water (Milli-Q, Millipore or the equivalent) and then fill the beaker up to 100 mL with methanol.
3. Carefully stir to mix but do not hit the cone.
4. Sonicate for 15 min by placing the beaker in sonicator bath.
5. Discard solvent appropriately.

Second Sonication:

1. Fill beaker with purified water (~80 mL).
2. Sonicate for 15 min as above.
3. Discard in sink and rinse with methanol.

Third Sonication:

1. Fill beaker with methanol.
2. Sonicate for 15 min as above.
3. Discard methanol appropriately and rinse with methanol as above.
4. Dry under stream of nitrogen (in hood).

## Figures and Tables

**Table 1:** Example instrument settings for the 6-plex assay.

Instruments	Parameters	Setting
Waters Quattro Micro, Acquity TQD and Xevo TQD	desolvation temperature (°C)	200
	source temperature (°C)	80
	capillary voltage (kV)	~3
	desolvation gas (L/hr)	1000
	cone gas (L/hr)	50

Sciex 3200 and 4000	CAD	2
	CUR	28
	IS (kV)	~3
	TEM (°C)	125
	GS1	30
	GS2	30
	ihe	on

**Table 2:** Multiple Reaction Monitoring Parameters.

**MRM Transition masses**	**Dwell time (sec)**	**Cone voltage (V)**	**Col. energy (V)**	**Compound ID**
384.30>264.20	0.050	15	18	GBA-P
391.30>271.30	0.050	15	18	GBA-IS
398.30>264.20	0.050	20	20	SMPD1-P
405.30>264.20	0.050	20	20	SMPD1-IS
412.30>264.20	0.050	16	18	GALC-P
417.30>264.20	0.050	16	18	GALC-IS
426.10>317.20	0.050	24	14	IDUA-P
431.20>322.20	0.050	24	14	IDUA-IS
484.20>384.10	0.050	22	14	GLA-P
489.20>389.10	0.050	22	14	GLA-IS
498.20>398.10	0.050	26	15	GAA-P
503.30>403.20	0.050	26	15	GAA-IS

**Table 3:** Autosampler and flow-injection conditions.

**Autosampler Settings**	
**Parameter**	**Setting**
Syringe Size	100 μL
Filling Speed	10 μL/s
Injection Speed	10 μL/s
Injection Volume	15 μL
Loop Size	10 μL
Loop Method	overfill
Wash Solvent	same as mobile phase
Wash Cycle	one wash with mobile phase to clean the syringe, then a second wash with mobile phase to clean the syringe and the injection value (total volume ~30-50 μL).

**Table 4:** Composition of the 6-plex assay cocktail.

**Component**	**Concentration**
GBA-S	500 μM
GALC-S	850 μM
GAA-S	350 μM
GLA-S	1200 μM
IDUA-S	250 μM
SMPD1-S	750 μM
GBA-IS	20 μM
GALC-IS	10 μM
GAA-IS	24 μM
GLA-IS	24 μM
IDUA-IS	15 μM
SMPD1-IS	15 μM
sodium taurocholate	28 mM
acarbose	8 μM
D-saccharic acid-1,4-lactone	40 μM
ZnCl2	0.6 mM
sodium oleate	4.27 mM
N-acetyl-galactosamine	50 mM
Succinic Acid	85 mM
sodium hydroxide	sufficient to bring pH to 4.71

**Table 5:** Ion chromatogram peak areas for the enzymatic products and internal standards^a^.

**Enzyme**	**Product**	**Internal Standard**	**Blank**^b^
GBA	200,000	200,000	1100
GAA	400,000	500,000	4500
GLA	600,000	600,000	6300
IDUA	120,000	200,000	1400
SMPD1	120,000	200,000	1700
GALC	100,000	100,000	1100

a Peak areas were obtained by integration of the multiple reaction monitoring ion chromatogram peaks using the Waters QuanLynx software. Shown are the approximate peak areas for a typical random newborn DBS using a Waters Acquity TQD mass spectrometer.

b Given are the integration of the multiple reaction monitoring ion chromatogram peaks for the enzymatic product peaks obtained when the 3 mm DBS punch was replaced with a 3 mm punch of filter paper (no blood control). Data are rounded to the nearest 10,000 (Product and Internal Standard) or 100 (Blank).
